# Mining Impacts on a Threatened and Burnt Swamp Ecosystem: Responses of the Microbial Soil Community

**DOI:** 10.1111/1758-2229.70397

**Published:** 2026-10-08

**Authors:** Nathali Machado de Lima, Xabier Vázquez‐Campos, Alexandria Thomsen, Mark Ooi, Miriam Muñoz‐Rojas

**Affiliations:** ^1^ Centre for Ecosystem Science, School of Biological, Earth & Environmental Sciences UNSW Sydney Sydney New South Wales Australia; ^2^ School of Biotechnology and Biomolecular Sciences The University of New South Wales Sydney New South Wales Australia; ^3^ Laboratorio de Biodiversidad y Funcionamiento Ecosistémico Instituto de Recursos Naturales y Agrobiología de Sevilla (IRNAS), CSIC Seville Spain

**Keywords:** functional composition, longwall mining, microbiome, soil biodiversity, wildfire

## Abstract

Peatlands are globally important carbon reservoirs that mitigate global warming while regulating biogeochemical cycles. However, these ecosystems are highly vulnerable to environmental disturbances, such as fire and mining. Despite the key ecological roles of soil microbes, descriptions of microbial assemblages in many peatland systems, and how they may shift under the dual disturbances of fire and mining, remain poorly addressed. Here, we assessed the composition and diversity of soil microbial communities in three mined and three unmined swamps in southeastern Australia, all affected by the unprecedented summer bushfires during 2019–2020. Bacterial and eukaryotic communities were analysed alongside soil chemical properties across a moisture gradient within each swamp. Our results revealed significant differences in communities due to mining activities, with the most pronounced differences observed within swamp communities rather than other sites along the moisture gradient. The eukaryotic community was significantly influenced by the combined effects of mining activity and fire history. The bacterial community was significantly correlated with pH. Our findings suggest that mining was associated with changes in eukaryotic diversity and bacterial community structure and functionality, with fire history potentially contributing to the observed patterns. This study underscores the critical, detrimental impacts that mining can have on peatland microbial composition.

## Graphical abstract

The evaluation of microbial communities and soil chemistry of three mined and three unmined swamps in the burnt southeastern Australia suggested that mining activities led to changes in the microbial community structure and functionality. This figure incorporates symbols sourced from BioRender and was created with BioRender.com.

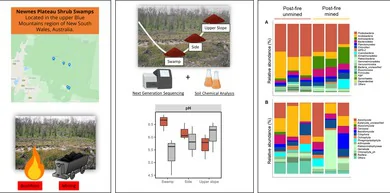

## 1 Introduction

Although peatlands occupy only 3% of the global land surface area, these systems represent a significant carbon store, having accumulated over 600 gigatons since the Holocene (Yu and Ehrenfeld 2010; Xu et al. 2018). These ecosystems are composed of up to 90% water and are characterized by low pH, limited dissolved oxygen, and low nutrient availability (Pemberton 2005). Their soils, commonly classified as organic soils, can reach depths of several meters or more (Pemberton 2005) and function as long‐term carbon sinks (Bu et al. 2011). Despite their ecological importance, peatlands are increasingly threatened by anthropogenic activities in addition to global warming, which can alter their hydrology and vegetation composition, leading to impacts on carbon dioxide and methane fluxes (Antala et al. 2022; Zhang et al. 2023).

The carbon balance in peat soils is closely linked with soil microbial processes (Miltner et al. 2012; Liang et al. 2017; Cong et al. 2020). Thus, changes in the soil microbiome of these communities can have a significant impact on carbon stability in the context of climate change (Zhao et al. 2021). Soil microbes have developed physiological and metabolic adaptations to thrive under the extreme conditions found in peatlands (Andersen et al. 2013). Functionally, these microbial communities play a critical role in the regulation of biogeochemical processes, including those of carbon, nitrogen, sulphur, phosphorus, and iron cycling, and may be more ecologically significant here than in other ecosystems (Lamers et al. 2012).

Peatland soil microbial communities are highly sensitive to environmental disturbances and have been used as bioindicators of ecological conditions (Sims et al. 2013a). Disturbances—both natural and anthropogenic—such as fire, water table alteration, nutrient deposition, rising temperatures, elevated CO_2_ concentrations, and peat extraction through mining, can significantly affect peatland ecosystems (Andersen et al. 2013). These impacts often result in shifts in soil microbial community composition, changes in microbial abundance, and modifications in microbial functions. For instance, drainage has been shown to reduce microbial richness and alter microbial functions (Birnbaum et al. 2023), while fire has resulted in the reduction of microbial biomass/abundance and induced community shifts (Hamman et al. 2007; Pressler et al. 2019; Huffman and Madritch 2018).

A common method for assessing ecological condition through microbial communities is the evaluation of the relative abundance of copiotrophic versus oligotrophic taxa, an indicator used to infer trophic status in wetlands and soils (Smit et al. 2001; Sims et al. 2013b). For instance, peatlands disturbed by urbanization were associated with a higher relative abundance of copiotrophic organisms (Christiansen et al. 2022).

In Australia, some peatlands are subject to disturbance both by fire and mining (Baird and Benson 2020). Fire is often a natural event, and plant communities maintain traits that allow persistence under particular fire regimes, mediated by soil moisture conditions that reduce fire severity. However, mining can lead to a major and persistent reduction in soil water retention capacity, which can directly impact plant and soil communities (Keith et al. 2020). Decrease of soil moisture can often amplify the severity of subsequent fires by allowing greater heat penetration into deeper soil layers, exacerbating negative effects on biodiversity and ecosystem services (Mason et al. 2021). Peatland recovery is sometimes possible, though it often occurs slowly over long periods (Anzooman et al. 2024).

The 2019–2020 Black Summer fires were notable for their extreme severity and scale, burning 10.3 million hectares of southern and eastern Australia (Penman et al. 2023). The duration, extent, intensity, and rate of fire spread during this period positioned these among the most severe on record (Keith et al. 2022), an event mirrored by similar recent record‐breaking mega‐fire events around the world (Bowman et al. 2017; Fidelis et al. 2018; Le Breton et al. 2022). Among the affected ecosystems, peatlands were considered to be ‘highly exposed’ to these bushfires (Keith et al. 2022), with potentially significant consequences for their structure and functional integrity (Fryirs et al. 2021). Keith et al. (2023) also suggested that mining activities could increase the susceptibility of peatlands to fire damage.

The composition of the plant community and the hydrology of peatlands are directly linked to the surrounding microbial community (Dimitriu et al. 2010; Yu and Ehrenfeld 2010), which plays a significant role in enhancing the overall biodiversity and ecological processes of the systems (Andersen et al. 2013). However, there is a large knowledge gap in both the description of microbial assemblages in many peatland systems and how these may shift under the dual disturbances of natural fire regimes and their interaction with mining. Peatlands are unique ecosystems and thus require specific research to guide conservation efforts (Pemberton 2005).

Here, we use an Australian case study focused on an ecosystem of peat‐accumulating palustrine wetlands, locally known as upland swamps (Keith et al. 2023), to assess the impacts of mining on the microbial community composition and soil chemical properties of burnt swamps. These swamps are subject to a natural regime of fire; however, longwall mining has impacted the hydrology of some swamps within the system, causing soil desiccation (Keith et al. 2023). Considering that abiotic soil properties (Dimitriu et al. 2010), plant community and hydrology (Yu and Ehrenfeld 2010) are all directly correlated with the surrounding microbial community, quantification of microbial community composition and function in swamps such as these would form a basis for future applied initiatives in conservation and restoration. Specifically, we set out to address the following research questions:

- What is the microbial and chemical composition of soils in these peatland swamp systems?
- Does composition differ between microsites along a moisture gradient within the swamps?
- Does composition differ between swamps, either impacted or not by longwall mining, and is there any indication of declines in their ecological condition?

We hypothesized that mined swamps would harbour a greater relative abundance of oligotrophic taxa, whereas the opposite was expected for pristine swamps, which commonly contain higher levels of nutrients. We expected to observe a greater impact at the centre of the swamp itself than at the margins, due to the substantial effect of mining on hydrology, and the role of water in regulating microbial dynamics.

## 2 Methods

### 2.1 Study Sites

All study sites (hereby referred to as Newnes Swamps) were in the Newnes Plateau, in the western Blue Mountains north of Lithgow (Benson and Baird 2012) ‐ NSW, Australia (Table 1). These swamps perform critical ecological functions, including the regulation of peak flow events, water purification (Hensen and Mahony 2010), and the provision of habitat for numerous threatened plant and animal species (Baird and Benson 2020). Despite its importance, the area has been threatened by many different external forces, like changes in its geomorphic structure (Cowley et al. 2018) and longwall mining processes (Baird and Benson 2020). Previous underground mining has resulted in hydrological changes (Keith et al. 2020). Newnes Swamps have previously been subject to fire events (Hammill and Tasker 2010), including after periods of severe drought, such as the 2019–2020 “Black Summer” bushfires (Baird and Benson 2020). While the fires burnt a significant area of the Newnes swamps, the impacts of fires in some areas were exacerbated by hydrological changes resulting from a history of longwall mining (Keith et al. 2020).

**TABLE 1:** Study sites located in the Newnes Plateau, in the western Blue Mountains north of Lithgow (NSW).

Site name	Latitude	Longitude	Land Status	Penultimate fire event date
Broad swamp	−33.3835	150.226	Unmined	> 20 years ago
Carne west swamp	−33.379	150.1981	Mined	> 20 years ago
Gang gang west swamp	−33.3805	150.2061	Mined	< 20 years ago
Happy valley swamp	−33.435	150.2216	Unmined	< 20 years ago
Sunnyside swamp	−33.3703	150.1817	Unmined	> 20 years ago
East Wolgan swamp	−33.3696	150.167	Mined	> 20 years ago

Annual rainfall on the Newnes Plateau is usually between 880 and 1080 mm (DEC 2006). Under the Köppen Climate Classification system, the study area is characterized as Temperate oceanic climate (Cfb) (Peel et al. 2007). We focused our sampling on the Newnes Plateau Shrub Swamps, which form part of the Temperate Highland Peat Swamps on Sandstone Endangered Ecological Community (Environment Protection and Biodiversity Conservation Act 1999). Newnes Plateau Shrub Swamps form narrow, elongated swamps which occur in low‐slope headwaters of the Newnes Plateau, in predominantly Triassic sandstone catchments, at approximately 900–1200 m elevation on deep sandy organic sediments that are permanently or periodically waterlogged (Benson and Keith 1990).

Newnes Plateau Shrub Swamp communities are dominated by shrublands of dense to open cover, with sedgelands replacing shrubs in wetter areas (Benson and Keith 1990). Floristic composition is greatly dependent on soil moisture gradients within the swamps and can also differ locally (Benson and Keith 1990) (Department of Environment and Heritage 2005). Margins of the swamps with surrounding woodlands have sharp boundaries with little intermixing of plant species. The slopes that surround the swamps are characterized by *Eucalyptus* forests and woodlands with a diverse shrub and herb layer (DEC 2006; Benson and Baird 2012).

### 2.2 Sampling Area

In January 2021, three unmined (Sunnyside, Happy Valley, Broad) and three mined swamps (East Wolgan, Gang Gang, Carne West) were selected for study (Table 1, Figure 1A). All study sites were burnt during the 2019–20 bushfires. To account for any variation in fire history between sites, we included time since the penultimate fire as a factor in our analysis. Fire history information was obtained from the NSW SEED data portal (https://www.seed.nsw.gov.au/; State Government of NSW and Department of Planning and Environment (DPE) 2010). Happy Valley swamp was disturbed due to an access track running through it, so this was noted when interpreting any results. At each site, soil samples were collected at three points along a moisture gradient from the center of the swamp itself (historically higher moisture), the swamp margin (hereafter referred to as the side), and 5–10 m above the swamp margin (hereafter referred to as the upper slope) (Figure 1B). We focused on the soil surface close to specific groups of plants common to the area (Department of Environment and Heritage 2005) to reduce bias from microbial community variation between plant species. Soil samples for side and upper slope samples were taken in proximity to *Leptospermum* species, including *L. trinervium* and *L. grandifolium*. At one site (Broad swamp), no *Leptospermum* species were found on the upper slope, and so samples were taken in the proximity of a *Eucalyptus* spp., a genus from the same family (Myrtaceae). Samples in swamp areas were taken in proximity to *Lepidosperma limicola*, except for one site (Happy Valley) where it was not present, and *Empodisma minus* was used instead.

**FIGURE 1:**
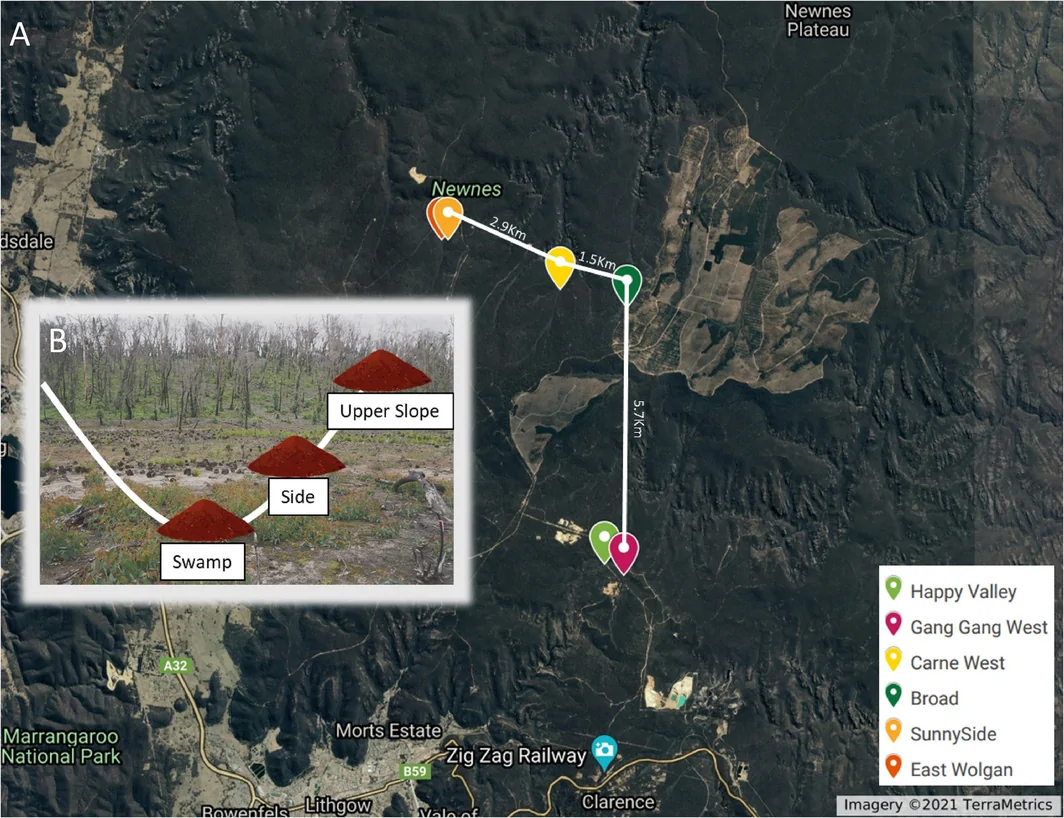
Location of the six studied swamps in the Newnes Plateau Shrub Swamps Area, NSW. Map created using Google Maps (A). Base map data 2021 Google; satellite imagery 2021 TerraMetrics. (B) Sampling microsites within each swamp (swamp, side and upper slope areas).

In each area, three composited soil samples (separated 10 m from each other) from the upper 3–5 cm were collected aseptically using a trowel. In total, we collected 3 samples × 3 areas × 6 swamps = 54 samples. The soil samples were kept in airtight bags and stored in a container with an ice pack (~4°C) until transported to the laboratory for further preparation. Not all the samples were used in the analysis. At the laboratory, samples were divided, with one subsample of each being stored at 4°C (1 sample × 3 areas × 6 sites = 18 samples), and another subsample maintained at −20°C for subsequent DNA extraction (1 sample × 3 areas × 6 sites = 18 samples).

### 2.3 Soil Microbial Analyses

To analyse the soil microbial community composition and diversity, we extracted DNA from each sample and performed amplicon sequencing. First, soil genomic DNA was isolated from 1 g of soil per sample using the DNeasy PowerSoil Kit (Qiagen, Venlo, Netherlands) according to the manufacturer's instructions. The DNA extracts were submitted to the Ramaciotti Centre for Genomics (UNSW, Australia) for two separate sequencing runs. The hypervariable regions V1‐V3 of the 16S rRNA gene were amplified using 27F (5′‐AGAGTTTGATCMTGGCTCAG‐3′) and 519R (5′‐GWATTACCGCGGCKGCTG‐3′) primers tailored with Illumina adaptor overhangs. Amplified products were purified and reamplified in a second indexing PCR, adding a unique Illumina Nextera XT index to each sample. Paired‐end amplicon sequencing was performed on the Illumina MiSeq platform using MiSeq Reagents kit v3 2 × 250 bp Nano. Similar procedures were applied to sequence the hypervariable region V9 of the 18S rRNA gene; instead, 1391F (5′‐GTACACACCGCCCGTC‐3′) and EukB (5′‐TGATCCTTCTGCAGGTTCACCTAC‐3′) primers were used, while afterward the paired‐end amplicon sequencing was performed on the Illumina MiSeq platform using 2 × 250 bp. Raw sequence data processing with OTU reporter as described in Machado de Lima et al. (2021), except for the taxonomic classification, which was performed to phylum and class levels (Tables S1 and S2) and assigned based on the classification given by SILVA database v132 (Quast et al. 2012).

### 2.4 Soil Chemical Analysis

Soil subsamples preserved at 4°C were dried at 35°C for 3 days, ground, and sieved through a 2‐mm mesh sieve. Total carbon, nitrogen, sulphur, and hydrogen were measured using a vario MACRO cube (Elementar, Sydney, NSW, Australia) at the Mark Wainwright Analytical Centre (UNSW Sydney). Soil pH was measured using 5 mg of the soil samples in 25 mL of deionized water. The mixture was shaken for 1 h, and pH quantification was performed using the Sper Scientific Benchtop Water Quality Meter 860,033.

### 2.5 Statistical Analysis

Statistical analyses were performed using R (v4.3.1; R Core Team 2015). Boxplots containing the relative abundance of the microbial community and the soil chemical component proportions were plotted using “ggplot2” (v3.4.3, Wickham et al. 2016). Differences in soil chemical composition, alpha diversity, and ratios between specific taxa among areas were tested using a linear model framework, while the “emmeans” function (“emmeans” package, v1.7.2) was applied to make comparisons (Lenth et al. 2019). The “phyloseq” package (v1.32.0, McMurdie and Holmes 2013) was used to subsample the number of reads per sample and create an object for downstream analysis. Following recommendations of Haegeman et al. (2013), Simpson and Shannon diversities were calculated, added by Simpson's evenness, using the function “alpha” available through the package “microbiome” (v1.10.0, Lahti and Shetty 2018). Differences between treatments were analysed within a linear model framework, followed by post hoc pairwise comparisons of estimated marginal means using the “emmeans” package in R. The effects of variables on the community were evaluated through a nonparametric permutational multivariate analysis of variance (PERMANOVA) using the function “adonis2” from the “vegan” package (v2.5‐7, Oksanen et al. 2019) on a Bray–Curtis dissimilarity matrix, with 999 permutations. The variables “area” and “land condition,” “area” and “fire history,” and “land condition” and “fire history” were tested (area × land condition; area × fire history; land condition *x* × fire history; land condition × fire history) jointly in the PERMANOVA. The variable “plant species” was only tested individually, while “area” and “land condition” were also tested individually. Although sample sizes were slightly unbalanced when considering the bacterial community in “mined” and “unmined” at the “swamp area” and “fire history,” PERMANOVA is robust to moderate imbalance.

The same matrix was used to generate a non‐metric multidimensional scaling (NMDS) analysis with the vegan function “metaMDS” to show the distribution of the community composition. Afterward, the function “betadisper” from “vegan” was used to test the homogeneity of variances. When necessary to check for the relative and shared contributions of different variables, the function “varpart” in the “vegan” package was applied, followed by “anova” to test for the significance of the pure effects. To identify significant soil chemical variables associated with microbial communities, we employed the “envfit” function from the “vegan” package.

### 2.6 Differentially Abundant Groups and Bacterial Functionality

MaAsLin2 v1.2.0 (Multivariate Association with Linear Models) was used to identify associations between mined and unmined areas (https://huttenhower.sph.harvard.edu/maaslin2). The functional prediction was performed with PICRUSt2 v2.4.1 without copy‐number normalization (Douglas et al. 2020). For the prediction, representative sequences were selected, based on abundance, from OTUs present in at least one sample at a relative abundance of 0.01%. NMDS was generated on the log‐transformed abundances of pathways in Euclidean space, with 10,000 permutations.

## 3 Results

### 3.1 Soil Microbial Community Composition and Diversity

The analysis of 16S rRNA gene amplicons resulted in a total of 48,653 operational taxonomic units (OTUs) after quality control, filtering, and applying the 97% threshold. This data was used to generate the relative abundance of each identified phylum (Figure 2). The samples contained from 9444 to 48,578 reads, which were subsampled at 20,901 reads per sample (samples below this threshold were excluded, resulting in the loss of one sample), summing to 28,736 OTUs that were utilized in all diversity analyses. A second run targeting the 18S rRNA gene and considering a clustering cut‐off of 97% resulted in 13,347 OTUs. Sample total reads varied from 4254 to 92,208 and were subsampled at 5316 (again resulting in the exclusion of one sample), summing to 4712 OTUs that were utilized in all diversity analyses.

**FIGURE 2:**
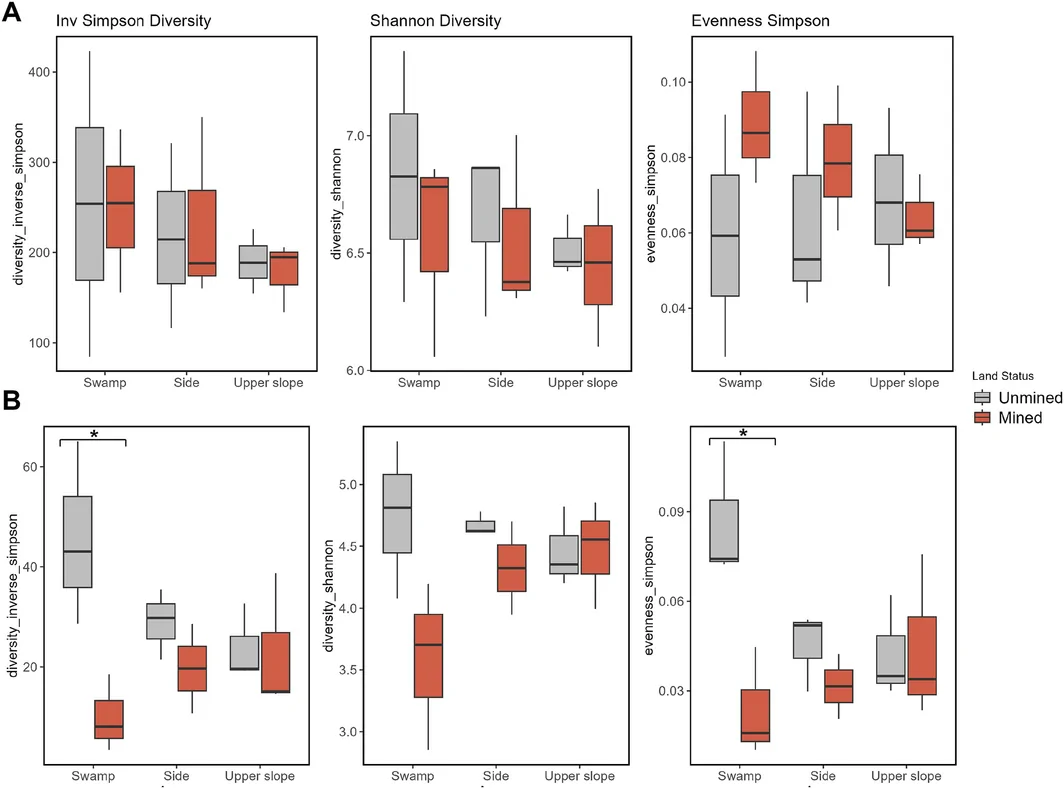
Alpha diversity between mined and unmined areas, based on Simpson and Shannon diversities and Simpson's evenness index. (A) Bacterial community, (B) Eukaryotic community. Asterisk (*) indicates significance levels at *p* < 0.005.

Mined swamps showed significantly lower eukaryotic alpha diversity (Inverse Simpson, estimate = 35.59 ± 9.67, *p* = 0.033) and Evenness (Simpson's evenness, estimate = 0.063 ± 0.017, *p* = 0.026) compared to unmined swamps, while alpha diversity in the side and upper slope areas was not significantly affected by mining. Similarly, bacterial diversity and evenness in all areas (i.e., swamps, side, and upper slope) did not differ between mined and unmined sites (Figure 2).

Regarding beta diversity, we found a significant difference in the bacterial community composition among the three areas analysed (*p* = 0.005, *R* ^2^ = 0.193). NMDS analysis highlighted these differences, showing that the bacterial communities on the side and upper slopes were more similar to each other than those in the swamps. PERMDISP considering areas also significantly pointed to heterogeneity in dispersion (*p* = 0.003). PERMANOVA analysis indicated a significant separation between mined and unmined areas (*p* = 0.041, *R* ^2^ = 0.09), which was also evident in the NMDS analysis. Swamp samples from mined sites remained highly separated from the unmined sites, while samples from the side and upper slope showed a smaller separation, indicating that they were less affected by mining (Figure 3). Beta diversity combining both variables (area and land condition) increases the explainability to 43% (*p* = 0.001). Conversely, the different eukaryotic community compositions were not associated with the different areas along with the moisture gradient or mining activity. However, pairwise analysis showed significant differences between swamps and upper slopes (*p* = 0.03, *R* ^2^ = 0.13).

**FIGURE 3:**
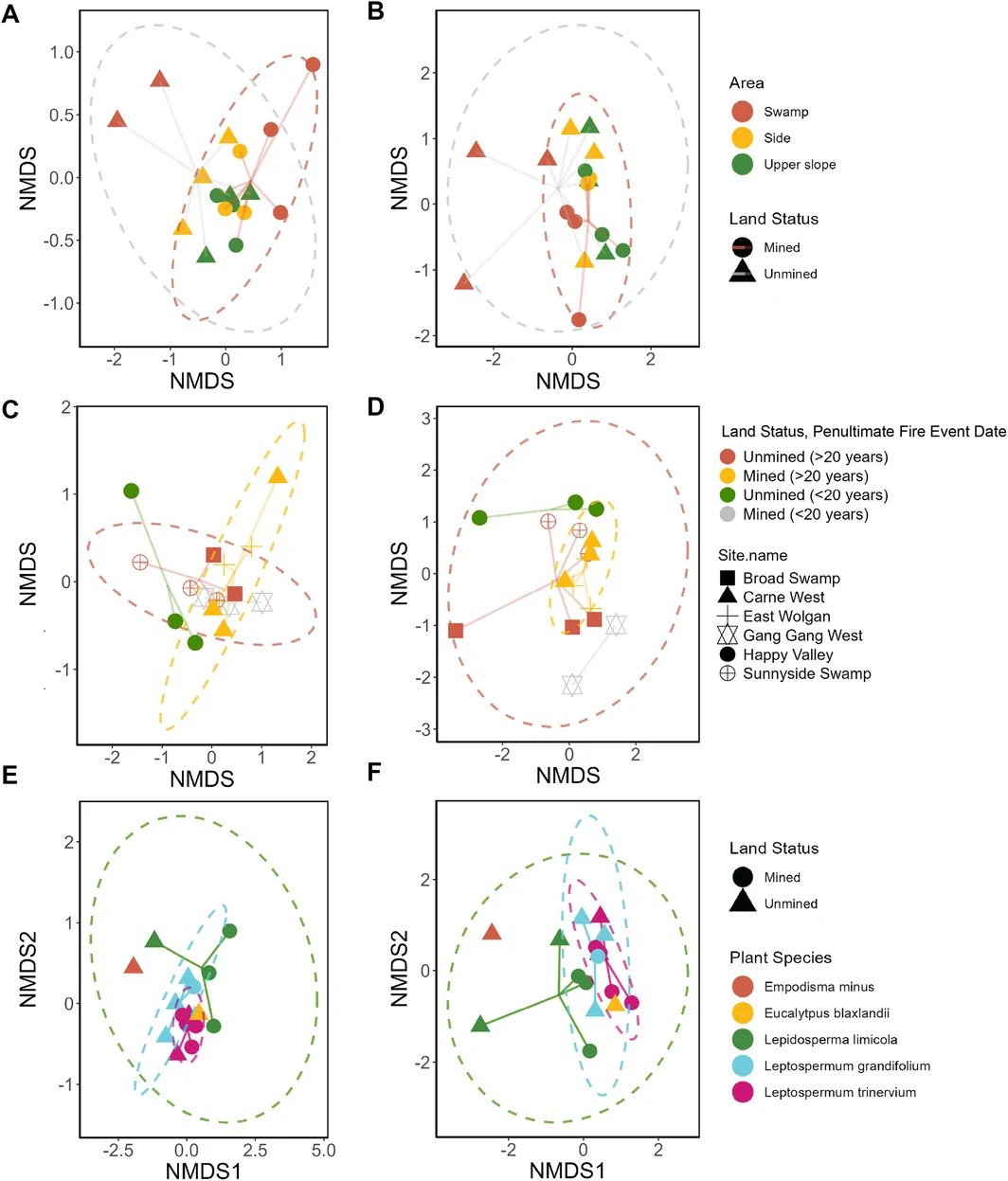
Non‐metric Multi‐Dimensional Scaling of the microbial community composition at the OTU level, based on Bray–Curtis dissimilarity. On the Left: Bacterial community—2D stress: 0.06. On the Right: Eukaryotic community—2D stress: 0.09. (A, B) Relation to Area and Land Status, including centroids calculated for Land Status. (C, D) Relation to the date of the penultimate fire events and Land Status, with centroids calculated for both variables combined; (E, F) Relation to the plant species from which the surrounding soil was sampled (with a centroid calculated for this variable) and Land Status.

Fire history alone did not significantly influence microbial community composition, as also indicated by PERMANOVA analysis (*p* > 0.05). Similarly, the combined effect of land condition and fire history did not significantly affect bacterial community composition (*p* = 0.1, *R* ^2^ = 0.22). However, NMDS analysis suggested greater differentiation between mined and unmined samples at sites where the penultimate fire event occurred less than 20 years ago than at sites where the penultimate fire occurred more than 20 years ago (Figure 3C). In contrast, eukaryotic community composition was significantly impacted by the combined effect of land condition and fire history (*p* = 0.02, *R* ^2^ = 0.24).

PERMANOVA and NMDS analyses also indicated that the plant species surrounding the soil sampling area played a role in shaping the soil microbial community composition (bacterial community: *p* = 0.002, *R* ^2^ = 0.364; eukaryotic community: *p* = 0.046, *R* ^2^ = 0.292). The three Myrtaceae plant species (*L. trinervium, L. grandifolium*, and *Eucalyptus* spp.) used for side and upper slope sampling were found to have similar microbial communities (Figure 3). However, the two swamp species used, *Lepidosperma limicola* and *Empodisma minus*, were found to have distinct microbial communities, while a separation within *Lepidosperma limicola* from distinct areas was also observed (Figure 3). Analysis of the variation partitioning in the bacterial community revealed that vegetation explained the largest proportion of variation, accounting for 10% of the total variance (adjusted *R* ^2^ = 0.1), whereas mining activity explained a smaller but measurable proportion of variation (6%, adjusted *R* ^2^ = 0.06). No shared fraction of explained variation was detected between mining and vegetation, indicating that their effects were statistically independent within the scope of this dataset.

Bacterial relative abundance based on the total OTU table showed that Proteobacteria, Acidobacteria, and Actinobacteria were the most abundant phyla in the Newnes Plateau Shrub Swamps (Figure 4). The ratios of copiotrophic to oligotrophic trait taxa, represented by Proteobacteria: Acidobacteria and Bacteroidetes: Acidobacteria were higher in the unmined swamps (Figures 4 and 5). Linear regression models applied by MaAsLin2 identified the bacterial taxa significantly associated with mining activities as Actinobacteria, Omnitrophicaeota, Spirochaetes, Bacteroidetes, Elusimicrobia, FCPU426, Nitrospirae, Rokubacteria, Verrucomicrobia, Acidobacteria, Gemmatimonadetes, and Deinococcus‐Thermus. Within the eukaryotic communities, only Annelida was significantly associated with mining activities (*p* < 0.05).

**FIGURE 4:**
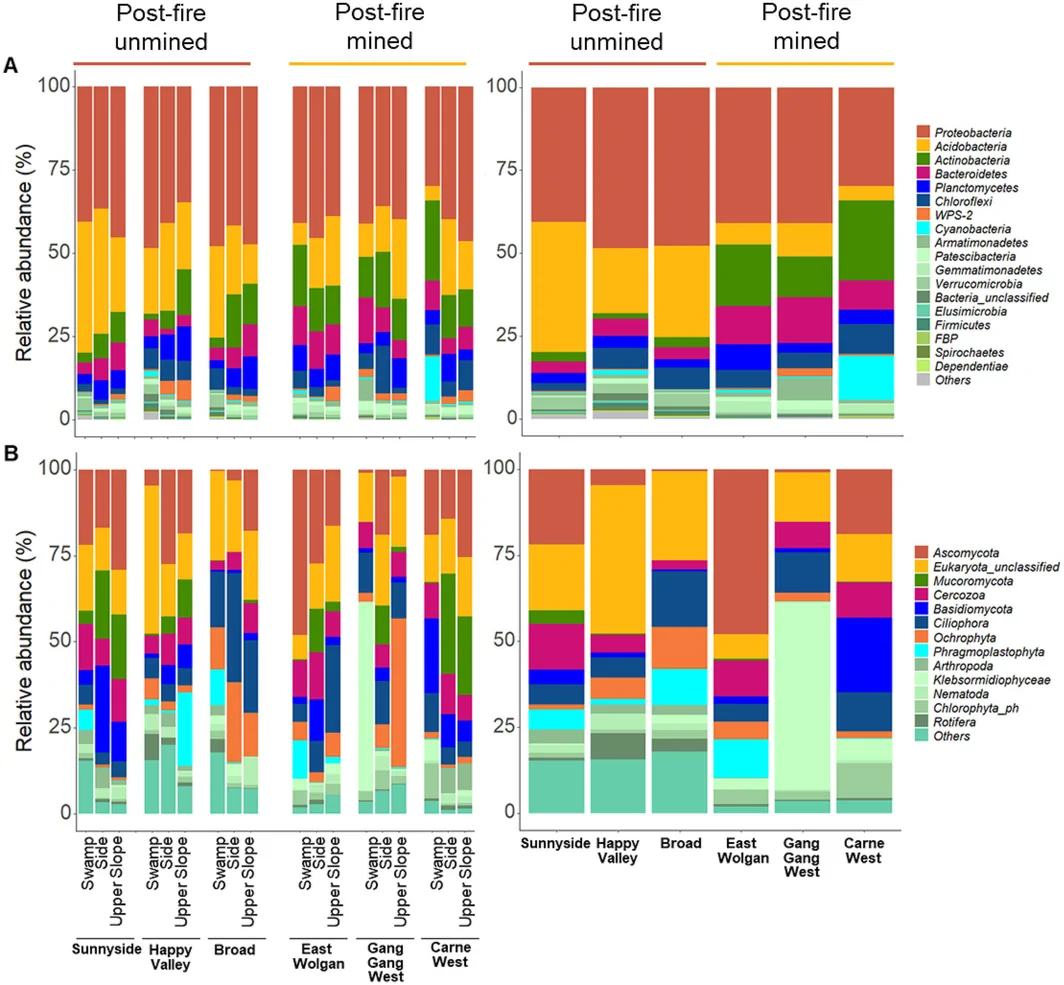
On the Left: Relative microbial abundance by proportion of gene sequence reads showing the assigned taxa in the three areas (swamp, side, upper slope) for each of the six locations. On the Right: Focus on the Swamp areas. (A) Bacterial relative abundance. (B) Eukaryotic relative abundance.

**FIGURE 5:**
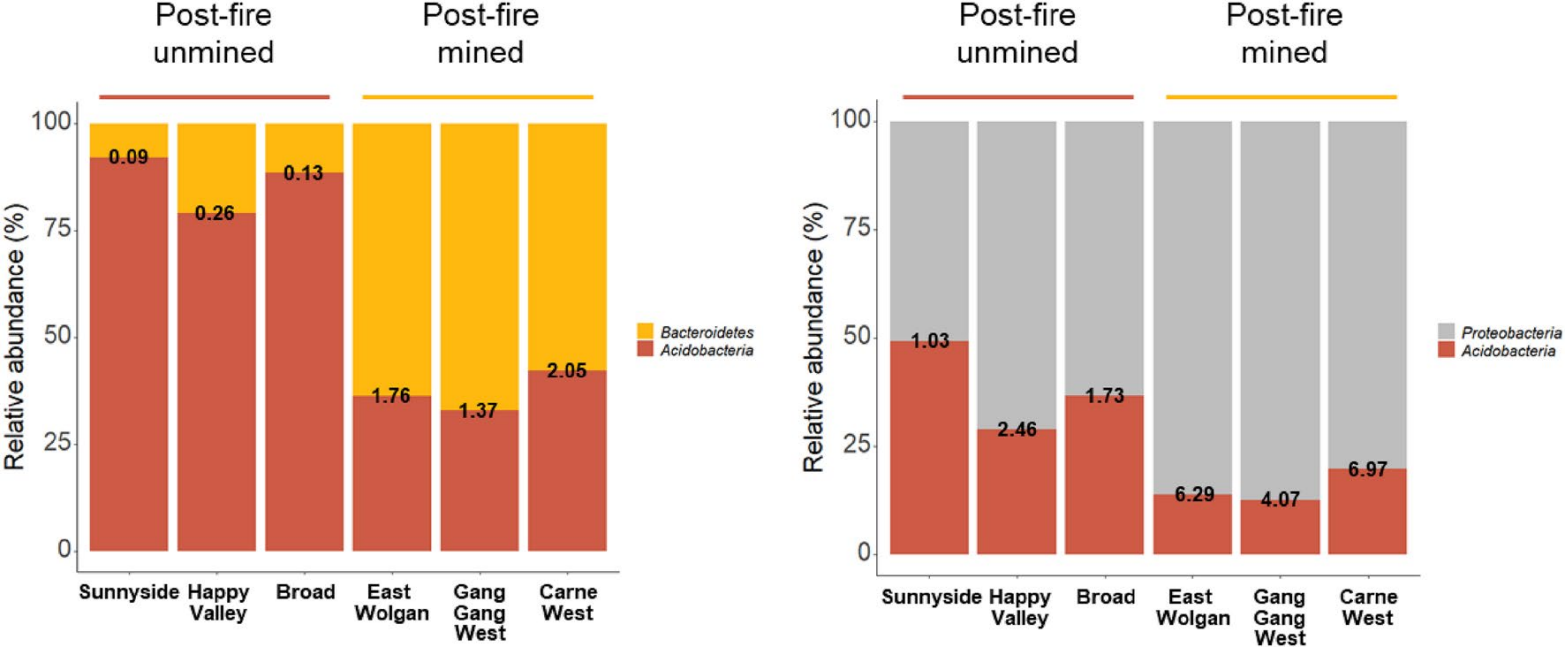
Relative microbial proportion of copiotrophic and oligotrophic trait taxa in the six different swamps. Numbers in the bars represent the actual ratios; Bacteroidetes:Acidobateria (on the right), Proteobacteria:Acidobacteria (on the left).

### 3.2 Soil Bacterial Functionality

Soil bacterial functional profiles were predicted using PICRUSt2 and based on the MetaCyc pathways per sample. Samples were distributed following a similar pattern to the community composition based on regular OTU counts (Figure 6). PERMANOVA analysis separated functionality among areas (*p* = 0.04, *R* ^2^ = 0.192); this effect may have been influenced by beta dispersion (Betadisper in the vegan package, ANOVA *p* < 0.01). PERMANOVA analysis also pointed out that bacterial functional profiles were significantly influenced by mining activities (*p* = 0.01, *R* ^2^ = 0.15), by mining and area together (*p* = 0.001, *R* ^2^ = 0.49), and time since the penultimate fire together with mining (*p* = 0.02, *R* ^2^ = 0.28). NMDS analysis also revealed a clear separation of swamps into distinct mined and unmined clusters, indicating a real difference in their functional structure after mining activities.

**FIGURE 6:**
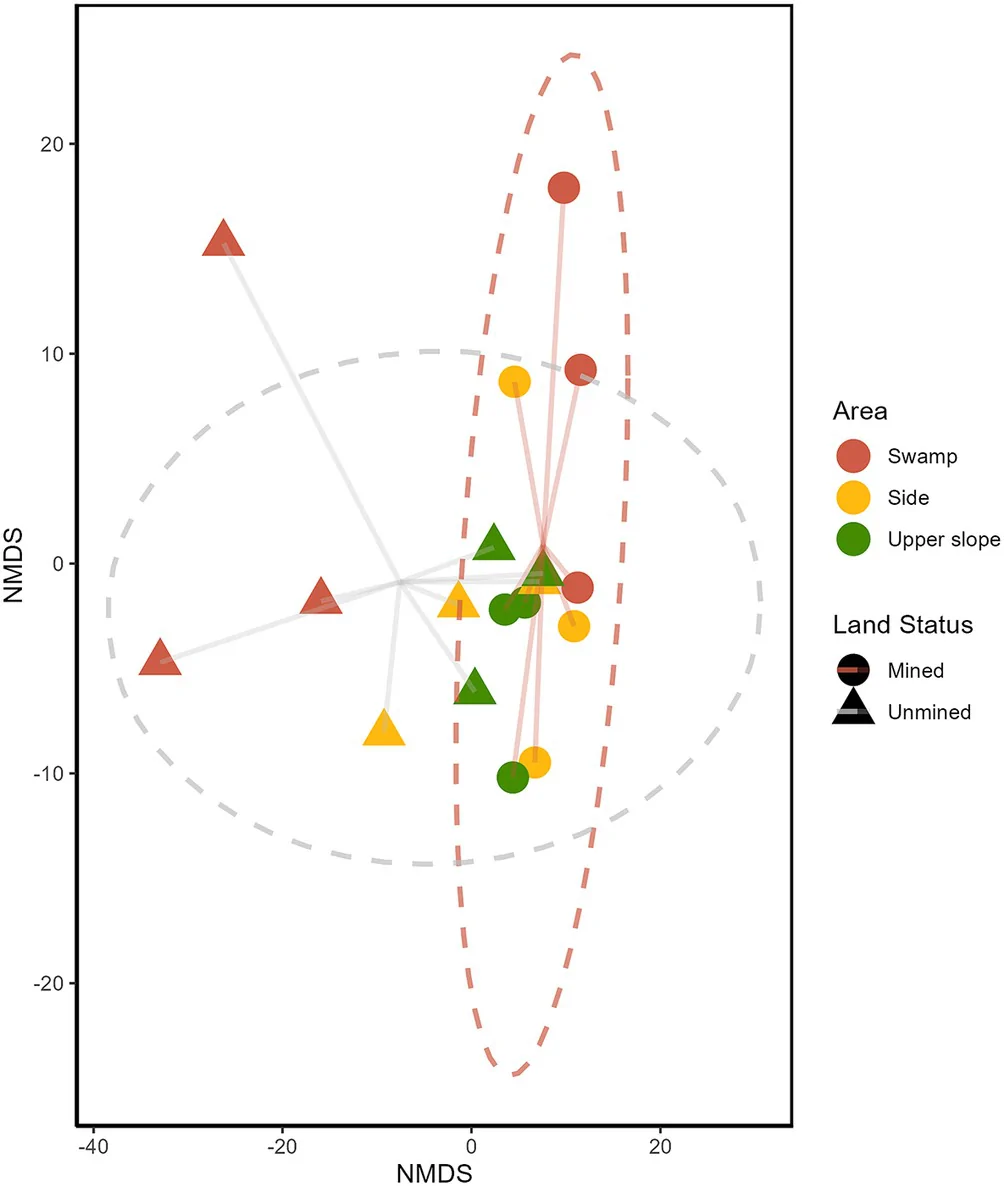
Functional differentiation of the bacterial communities of three areas, swamp, side and upper slope showed in an NMDS ordination based on MetaCyc pathways determined using PICRUSt2 v2.2.0‐2D stress: 0.08. Including centroids calculated for Land Status.

### 3.3 Soil Chemical Variables

Our analysis did not find differences in the chemical components across the side and upper slope when using mining or the date of the penultimate fire event as an indicator. However, when removing the Happy Valley site from the analysis, swamps from Broad and Sunnyside sites (unmined) showed significantly higher total carbon (estimate = −16.2 ± 2.85, *p* = 0.011), nitrogen (estimate = −0.717 ± 0.159, *p* = 0.02), and hydrogen (estimate = −2.161 ± 0.59, *p* = 0.036) when compared to the mined swamps. Soil pH values were low in the swamps, ranging from 4.5 to 5.8, with a slight increase in the mined areas, ranging from 6.7 to 6.9 (Figure 7). The “envfit” function test showed a significant correlation between pH and the total bacterial community distribution (*p* = 0.014, *R* ^2^ = 0.453). Sulphur was associated with total eukaryotic community distribution (*p* = 0.034, *R* ^2^ = 0.492).

**FIGURE 7:**
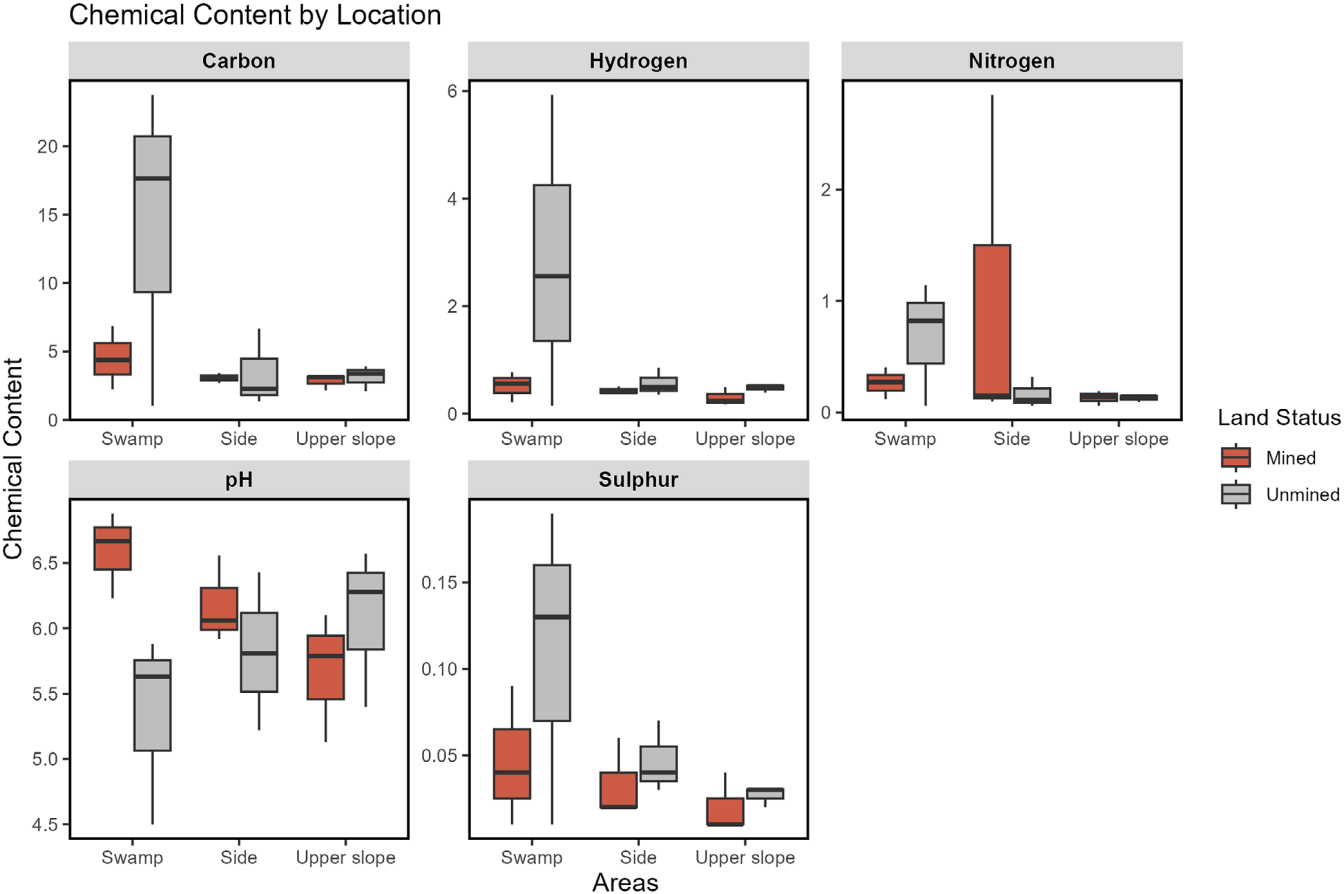
(From left to right, top to bottom) Boxplots of carbon (%), hydrogen (%), nitrogen (%), pH, and Sulphur (%) measurements across the three areas: swamp, side, upper slope.

## 4 Discussion

Our findings indicate that mining was significantly associated with soil bacterial community composition and predicted functional profiles, with stronger effects observed when mining was considered together with sampling area. In contrast, eukaryotic community composition was not significantly associated with mining alone, although eukaryotic diversity was lower in mined swamps. Notably, the combined effect of land condition and fire history was significantly associated with eukaryotic community composition, suggesting that the response of eukaryotic communities to mining may depend on fire history. Plant species was also significantly associated with both bacterial and eukaryotic community composition. Overall, these findings indicate that bacterial and eukaryotic communities responded differently to the environmental factors examined, with bacterial communities showing a clearer association with mining, whereas eukaryotic communities showed a stronger response when mining and fire history were considered together. These findings are consistent with previous studies showing that mining and other disturbances can substantially alter soil microbial communities in peatland ecosystems (Andersen et al. 2013; Young 2017; Reumer et al. 2018).

Proteobacteria and Acidobacteria were the dominant bacterial phyla in the unmined swamp samples. These phyla are commonly reported in peatlands, usually presenting in inverse proportion to each other, and are related to changes in pH and substrate availability (Smit et al. 2001; Hartman et al. 2008; Urbanová and Bárta 2014). It has been suggested that the ratio between Proteobacteria and Acidobacteria can be used as a broader indicator of the soil ecosystem nutritional status, with low ratio values usually indicating oligotrophic conditions (Smit et al. 2001). It is important to highlight that this indicator relies only on the phylum level, being very inaccurate; however, it is still valid when discussing carbon availability (Bai et al. 2017). Acidobacteria is considered the dominant bacterial phylum in nutrient‐poor environments (Urich et al. 2008), and the phylum also appears to play a role in cellulose decomposition within acidic peat environments (Pankratov et al. 2011). Despite the high abundance of Acidobacteria in our unmined swamps and its expected oligotrophic signature, the carbon content in these areas was high. This source of carbon is generally not accessible, though, being formed by recalcitrant compounds due to anoxic and acidic conditions (Elliott et al. 2015). The increase in Proteobacteria and Bacteroidetes may represent a stage where the microbial community is particularly efficient in utilizing available carbon sources. Higher Proteobacteria: Acidobacteria and Bacteroidetes: Acidobacteria ratios observed in the mined swamps suggest a microbial response to disturbance, likely leading to increased carbon metabolism and its release from the soil in the form of CO_2_ (Cowley and Fryirs 2020; Christiansen et al. 2022). Despite the copiotrophic phylum signature in the mined swamps, total carbon content was relatively lower; however, as indicated by other studies, Newnes mined swamps were drained (Baird and Benson 2020; Keith et al. 2022), and drainage may increase the labile pool, microbial activity, and carbon mineralization (Kalisz and Lachacz 2023). Notably, drainage increases oxic conditions and facilitates microbial decomposition of stable carbon, leading to reduced effective carbon accumulation (Freeman and Kang 2001; Andersen et al. 2013; Kalisz and Lachacz 2023). PICRUSt2 analysis suggested potential functional differences between mined and unmined swamps, consistent with observed shifts in community composition and trophic status. However, functional inferences derived from this approach have known limitations (Corrigan et al. 2015) and should be interpreted cautiously and used only as complementary evidence.

Soil pH was low for all studied sites, which seems to be a natural condition for the area, already reported in previous studies (Christiansen et al. 2019). Unmined swamps exhibited lower pH values compared to the mined ones, but differences were not significant. This can be attributed to the limited sample size in the analysis. Fitting environmental variables using envfit reaffirmed the importance of soil pHin driving bacterial distribution in the studied areas (in this case, the analysis incorporated a larger dataset, encompassing not only swamp samples but also samples from other microsites). Frequently highlighted as the best predictor of bacterial community composition in soil microbial communities (Bååth and Anderson 2003; Lauber et al. 2009), soil pH is also widely known as a strong variable influencing microbial distribution in wetlands (Lauber et al. 2009). Actinobacteria are another group that has a high acid tolerance response (ATR), and this may explain their high abundance in Sunnyside and Broad swamps, as well as their slightly lower abundance in Happy Valley swamp (pH 5.88), and their drastically lower abundance in the mined swamps (pH values > 6.0). Many other groups of bacteria are also limited by pH (Lauber et al. 2009), which also may justify the greater bacterial diversity and the proliferation of other groups (such as Actinobacteria and Bacteroidetes) in many mined swamps.

Vegetation had a broad impact on the soil bacterial and eukaryotic community, reinforcing that plants control their root systems and surrounding environment through rhizosphere resources, such as root exudates, allelochemicals, and soil nutrients (Chroňáková et al. 2019; Proctor and He 2021). This effect was separated from the effect of mining, since their shared contributions were discarded by the variation partitioning analysis. Plant species on the side and upper slope exhibited significant similarity in their microbial communities, whereas the same *Lepidosperma limicola* in various swamps displayed distinct microbial communities, influenced by differences in soil chemistry and mining activity. This suggests that while plants visibly influence their rhizospheric microbial communities, the ultimate composition is influenced by other variables (Zhang et al. 2023).

In contrast to the bacterial community, we did not find a significant influence of mining on the eukaryotic community at the OTU level. Among the eukaryotic taxa analyzed, only Annelida showed a significant difference in abundance between mined and unmined sites. However, eukaryotic alpha diversity was significantly lower in mined swamps, and NMDS ordination showed differentiation between mined and unmined swamp samples. Together, these results suggest that mining may affect some aspects of the eukaryotic community despite the absence of a significant overall effect on community composition. The lack of a significant effect on overall community composition may partly reflect the high variability observed among eukaryotic samples, including among swamps under similar conditions. In contrast, when mining was considered in combination with fire history, a significant association with eukaryotic community composition was detected. Previous studies have shown that fungal communities can be particularly sensitive to fire and may fail to recover to their pre‐fire state even after 30 years (Meng et al. 2021). In our study, NMDS ordination showed greater differentiation between mined and unmined samples at sites where the penultimate fire occurred less than 20 years ago. However, the temporal sequence of mining and fire events limits the interpretation of this relationship. In particular, the 2013 fire preceded the establishment of mining operations at Gang Gang West Swamp (2015–2016; Baird and Benson 2020). Consequently, we cannot conclude that mining exacerbated the effects of fire. Rather, our results indicate that eukaryotic community composition was significantly associated with the combined effects of mining and fire history, without allowing the direction or causality of this relationship to be determined.

We observed a difference in the soil chemical variables between mined and unmined swamps when the Happy Valley swamp area was excluded from the analysis. We made this decision because of a confounding factor, a track across the Happy Valley swamp that may have influenced the nutrient content in the area we sampled. This track had led to the runoff of sediments, affecting the specific location of our sampling. Excluding this sample weakened the robustness of the inference; significant differences between soil carbon, nitrogen, and hydrogen contents were still observed. Clear differences were also observed for the soil pH measurements, with this variable being significantly related to the bacterial community distribution. These differences are usually correlated with extreme or repeated fire events. Increases in soil pH can be related to the production of ash and its alkaline hydrolysis effect (Meng et al. 2021), while depletions in carbon and nitrogen can be driven by losses of plant biomass inputs and reduced potential decomposition (Pellegrini et al. 2020). Fungi present a weaker direct connection with pH, exhibiting a wider pH range for optimal growth (Rousk et al. 2010). This explains why the eukaryotic community distribution in this study was not related to pH, but instead to differences in Sulphur.

Our findings, particularly the significant association between eukaryotic community composition and the combined effects of mining and fire history, added by the pronounced differences in soil chemistry, especially pH, suggest that mining may have altered the environmental conditions that shape microbial responses to disturbance. This could potentially influence how these ecosystems respond to fire; however, this interpretation remains speculative because our dataset did not include unburned sites that were not affected by the 2019–2020 bushfires. Furthermore, the temporal relationship between mining activities and previous fire events prevents us from confidently disentangling their individual effects and establishing a causal link. In particular, at Gang Gang West Swamp, the penultimate fire occurred in 2013, prior to the establishment of mining operations in 2015–2016 (Baird and Benson 2020). Therefore, the significant combined effect of mining and fire history cannot be interpreted as evidence that mining exacerbated the effects of fire. Rather, our results suggest a relationship between these disturbances, whose direction and underlying mechanisms cannot be determined from the present dataset. The hypothesis of “context‐dependent effect,” whereby mining activities make swamps more prone to the influence of fire, is consistent with patterns reported by Keith et al. (2023) that showed that the undermined swamps experienced a shift in their hydrological regime shortly after the extraction of the coal seam, making the swamps more susceptible to significant fire impacts. Also, previous observations have shown that the soil surface was unnaturally dry in the undermined swamps and that the vegetation in those areas was negatively impacted to a greater degree (Baird and Benson 2020; Keith et al. 2022). The fact that we observed bigger differences in microbial communities between previously mined and unmined areas at the center of the swamp itself than at the margins also reinforces the potential strong effect of hydrology.

Anzooman et al. (2024) used vegetation‐normalized Difference Vegetation Index (NDVI) and Soil Moisture Index (SMI) to assess Newnes Plateau post‐fire recovery patterns. The authors observed that the mined swamps were not dried; instead, they had lower soil moisture before the fire event. Still, the authors suggested that recovery was influenced by post‐fire hydrology, with higher soil moisture resulting in faster recovery. This same study also stated that their analysed undermined swamps showed vegetation recovered by 2022. However, the study relied solely on NDVI and was validated using field vegetation surveys from a single site. While NDVI effectively captures vegetation greenness, it may overlook critical functional aspects of ecosystem recovery (Buma 2012). We sampled our soils in January 2021; therefore, future sampling should be considered to further investigate soil microbial communities after potential mid‐ or long‐term vegetation recovery.

## 5 Conclusion

Here, we evidence that longwall mining can have substantial impacts on the soil microbial communities of peatland systems, exacerbating the negative effect of fire on swamps such as Newnes. These impacts can potentially lead to the deterioration or eventual collapse of these fragile ecosystems, as previously observed in other upland swamps (Young 2017). Higher abundances of taxa associated with increased carbon metabolization suggest a potential release of carbon from the soil in the form of CO_2_ (Christiansen et al. 2022). This can contribute to the possible transformation of these systems from being a carbon sink to becoming a carbon source (Cowley and Fryirs 2020).

It is important to note that our investigation was carried out during a short period following the 2019 bushfires, and many variables may change over the coming years. Therefore, ongoing research is necessary to determine how the impacts on soil and microbial composition will evolve over time. Sequential analyses are particularly critical for accurately predicting the long‐term consequences of mining and fire on the Newnes Plateau Shrub Swamps, as well as their resilience and risk reduction (Fierer 2017). Furthermore, the microorganisms identified in this study may have potential applications in future restoration strategies, such as bioinoculants and bioindicators.

## Author Contributions

**Mark Ooi:** conceptualization, investigation, funding acquisition, writing – review and editing, supervision. **Alexandria Thomsen:** conceptualization, investigation, writing – review and editing. **Miriam Muñoz‐Rojas:** supervision, conceptualization, investigation, funding acquisition, writing – review and editing. **Nathali Machado de Lima:** conceptualization, investigation, data curation, writing – review and editing, writing – original draft, validation, visualization. **Xabier Vázquez‐Campos:** writing – review and editing, formal analysis, data curation.

## Funding

This work was supported by the Ministerio de Ciencia e Innovación (PID2021‐123097OA‐I00 and PID2024‐161692OB‐C33) and the Australian Research Council (LP180100741).

## Conflicts of Interest

The authors declare no conflicts of interest.

## Supporting information


**Table S1:** Relative abundance (%) of bacterial classes per sample, including information on area, date of the penultimate fire events, and land status.

**Table S2:** Relative abundance (%) of fungal class per sample, including information on area, date of the penultimate fire events, and land status.

## Data Availability

The data that support the findings of this study are openly available in National Center for Biotechnology Information at https://submit.ncbi.nlm.nih.gov/subs/bioproject/SUB14380744/overview, reference number BioProject PRJNA1102035.
